# Organic synthesis using photoredox catalysis

**DOI:** 10.3762/bjoc.10.107

**Published:** 2014-05-12

**Authors:** Axel G Griesbeck

**Affiliations:** 1 University of Cologne, Department of Chemistry, Organic Chemistry, Greinstr. 4, D-50939 Köln, Germany; Fax: +49 (221) 470 5057

**Keywords:** photoredox catalysis

Natural photosynthesis is a remarkable chemical machinery that enables our life on earth and delivers a constant stream of oxygen and organic biomass. We should acknowledge this fact with humbleness, especially because we have not been able yet to mimic this process in a reliable way even after decades of intense research. The basic mechanistic principle behind photosynthesis is photoredox catalysis or light-driven charge separation, which leads to an energy harvesting process by taking advantage of the reduction products and filling the holes by a sacrificial electron donor, water. Fortunately, we can use the waste product from this process, oxygen, for breathing.

For applications in organic synthesis, the principles of photoredox chemistry serve as guidelines, i.e., photoinduced electron transfer (PET) kinetics and thermodynamics as expressed in the Rehm–Weller and Marcus equations. For catalytic versions, the photoinduced redox processes require efficient and robust photocatalysts, and in many cases appropriate sacrificial components. In recent years, three major groups of light-absorbing molecules/materials have been (re)investigated, which facilitate a wide range of redox activation from their excited states: transition metal complexes (e.g., the thoroughly investigated Ru(bipy)_3_ and other Ru or Ir complexes) with strong MLCT transitions, organic dyes such as xanthene, porphyrine or phthalocyanine dyes (e.g., eosin Y), and colloidal semiconductor particles (e.g., TiO_2_) [1–9]. In addition, combinations of light-absorbing materials have been studied such as dye-coated semiconductor nanoparticles. On the substrate side, the focus is on redox-active donor/acceptor molecules, which range from all kind of aromatic, olefinic and carbonyl-type electron acceptor compounds to heteroatom-linked electron donors. The relevance of carbon–carbon bond formation for organic synthesis is also depicted in these processes, and in recent years, enantioselective versions of these processes as well as unusual activation and coupling modes have been developed. In contrast to the “traditional” catalysis areas such as metal-, organo- and biocatalysis, photoredox catalysis (and photocatalysis in general) is a young research field with regard to synthetic applications. The collection of papers in this Thematic Series on organic synthesis using photoredox catalysis shows this convincingly.

It was a great pleasure to act as the editor of this Thematic Series on photochemical reactions, and I would like to thank all authors for their excellent contributions and the staff of the Beilstein-Institut for their professional support.

Axel G. Griesbeck

Cologne, April 2014
